# The Association Between Type 2 Diabetes Mellitus and Melanoma Prognosis in Patients With High‐Risk Primaries

**DOI:** 10.1111/ajd.14605

**Published:** 2025-09-24

**Authors:** Hansa Sharma, Maria Celia B. Hughes, Danielle Gavanescu, B. Mark Smithers, Kiarash Khosrotehrani, Lena von Schuckmann

**Affiliations:** ^1^ Dermatology Research Centre Frazer Institute, The University of Queensland Brisbane Queensland Australia; ^2^ Queensland Melanoma Project, Princess Alexandra Hospital The University of Queensland Brisbane Queensland Australia; ^3^ Department of Dermatology Princess Alexandra Hospital Brisbane Queensland Australia; ^4^ Department of Dermatology Sunshine Coast University Hospital Sunshine Coast Queensland Australia

**Keywords:** dermatology, epidemiology, melanoma, prognosis, skin neoplasms, type 2 diabetes mellitus

Melanoma is a potentially aggressive skin cancer, and the determinants of its prognosis are yet to be fully understood [1]. Several studies have found type 2 diabetes mellitus (T2DM), a chronic metabolic disorder, to be linked to an increased risk of melanoma onset, thicker and/or ulcerated tumours, and an increased risk of recurrence, though some studies have shown conflicting results [2, 3, 4].

Our prospective cohort study recruited 700 eligible patients aged over 16 with newly diagnosed T1b–T4b (using AJCC 8th edition) cutaneous melanoma from various public and private healthcare facilities in Queensland, Australia between 2010 and 2014. We defined ‘high‐risk’ melanoma as T1b and above, > 0.8 mm or < 0.8 mm with ulceration. Patients found with macroscopic lymph node metastasis or distant metastatic disease within 30 days of diagnosis were excluded. Further details of the study have been published previously [4]. This study was approved by the respective ethics committees.

A baseline questionnaire collected information on participants' age, sex, body mass index (BMI) [5], diabetes status, smoking status, statin usage in the preceding 5 years, and socioeconomic status (SES; calculated using the Socio‐Economic Index for Areas [SEIFA] [6]). Tumour thickness and ulceration were extracted from the participants' histopathology reports. Recurrence data was obtained during follow‐up, from hospital records and from the Queensland Cancer Registry. Multiple recurrences within 30 days were considered one event and assigned the earliest date of recurrence. Follow‐up times were calculated as the number of months from the date of diagnosis to the date of first recurrence, death, last communication/withdrawal, or 84 months, whichever came first.

Tumour thickness was grouped as thinner (≤ 2.0 mm) and thicker (> 2.0 mm). Patient's socioeconomic status was categorised as lower (deciles 1–6) or higher (deciles 7–10), using the Index of Relative Socio‐Economic Advantage and Disadvantage (IRSAD) [6]. Univariable chi‐squared tests (*p* < 0.05) were used to describe patient and tumour characteristics with diabetes status. We used logistic regression and Cox proportional hazards to associate diabetes status with tumour thickness and 7‐year recurrence, respectively. We adjusted for age, sex, presence of ulceration, smoking status, statin use, and tumour thickness (where relevant). Sub‐group analyses were conducted using SES and BMI. Adherence to proportionality was verified using a Kolmogorov‐type supremum test. Participants who were recurrence‐free and alive (*n* = 460), died without having a recurrence (*n* = 44), or were lost to follow‐up (*n* = 17) by 7 years were censored. All analyses were performed using SAS version 9.4 (SAS Institute Inc., Cary, NC).

Of the 700 study participants, 94 (13%) had a diagnosis of T2DM at the time of melanoma diagnosis. Participants with T2DM were more commonly male (67%), had an obese (> 30 kg/m^2^) BMI (59%), used statins (63%), and had a lower SES (60%) (Table 1).

**TABLE 1:** Patient and tumour characteristics of cohort in relation to diagnosed type 2 diabetes mellitus (T2DM).

	Diagnosed with diabetes			*p*
	Total	No	Yes	
	*n* (%)	*n* (%)	*n* (%)	
	700 (100)	606 (87)	94 (13)	
Age (years)				
< 55	189 (27)	183 (30)	6 (6)	**< 0.01**
55– < 70	286 (41)	243 (40)	43 (46)	
≥ 70	225 (32)	180 (30)	45 (48)	
Sex				
Male	410 (59)	347 (57)	63 (67)	0.07
Female	290 (41)	259 (43)	31 (33)	
BMI (kg/m^2^)				
< 25	178 (27)	168 (30)	10 (11)	**< 0.01**
25.0–30.0	264 (41)	238 (43)	26 (30)	
> 30	204 (32)	153 (27)	51 (59)	
Thickness (mm)				
≤ 2.0	433 (62)	373 (62)	60 (64)	0.67
> 2.0	267 (38)	233 (38)	34 (36)	
Presence of ulceration				
No	504 (72)	439 (72)	65 (69)	0.51
Yes	196 (28)	167 (28)	29 (31)	
Statin use				
No	484 (69)	449 (74)	35 (37)	**< 0.01**
Yes	213 (31)	154 (26)	59 (63)	
Smoking status				
Never smoked	351 (53)	311 (54)	40 (43)	0.06
Current smoker	54 (8)	45 (8)	9 (10)	
Former smoker	263 (39)	219 (38)	44 (47)	
Recurrence				
No	521 (74)	455 (75)	66 (70)	0.31
Yes	179 (26)	151 (25)	28 (30)	
Socioeconomic status^a^				
Lower	358 (51)	302 (50)	56 (60)	0.08
Higher	342 (49)	304 (50)	38 (40)	

*Note:* For some variables, the summed total is less than the total number of patients due to missing values. Bold values indicate *p* < 0.05.

Abbreviation: BMI, body mass index.

^a^ Calculated using the Socio‐economic Index for Areas and the Index of Relative Socio‐economic Advantage and Disadvantaged (SEIFA‐IRSAD). Lower = deciles 7–10; Higher = deciles 1–6.

We found no significant difference in the likelihood of having a thicker melanoma at diagnosis between patients with and without T2DM (adjusted odds ratio [OR] 0.66, 95% confidence interval [CI] 0.40–1.09, *p* = 0.10). We found that lower SES patients with T2DM were significantly less likely to have a thicker melanoma than their non‐diabetic counterparts (OR 0.49, 95% CI 0.25–0.94, *p* = 0.03) (Table 2). Assuming that lower SES individuals with T2DM, compared to those without, have more consistent access to healthcare, this result may be due to opportunistic lesion detection during diabetes‐related health checks. Additionally, this result could be due to an alternate health behaviour or medication use not captured in our study. Further evidence from large population‐based cohorts could help clarify these results.

**TABLE 2:** Association between tumour thickness at diagnosis and type 2 diabetes mellitus (T2DM) using logistic regression.

		*N* (%)	Unadjusted OR^a^ (95% CI)	*p*	Adjusted^b^ OR (95% CI)	*p*
Diabetes						
No		233 (87)	1.00 *Reference*		1.00 *Reference*	
Yes		34 (13)	0.91 (0.58–1.43)	0.67	0.66 (0.40–1.09)	0.10
Stratified analyses						
Socioeconomic status^c^						
Lower	No Diabetes	132 (87)			1.00 *Reference*	
	Diabetes	20 (13)			0.49 (0.25–0.94)	**0.03**
Higher	No Diabetes	101 (88)			1.00 *Reference*	
	Diabetes	14 (12)			0.99 (0.46–2.15)	0.98
BMI^d^						
Normal weight	No Diabetes	62 (93)			1.00 *Reference*	
	Diabetes	5 (7)			1.66 (0.40–6.96)	0.49
Overweight	No Diabetes	95 (90)			1.00 *Reference*	
	Diabetes	10 (10)			0.73 (0.29–1.82)	0.49
Obese	No Diabetes	57 (78)			1.00 *Reference*	
	Diabetes	16 (22)			0.63 (0.30–1.33)	0.22

*Note:* Bold values indicate *p* < 0.05.

Abbreviations: BMI, body mass index; CI, confidence interval; OR, odds ratio.

^a^ Odds ratio of a thicker (> 2.0 mm) tumour at diagnosis.

^b^ Adjusted for age, sex, presence of ulceration, smoking status, and statin use.

^c^ Calculated using the Socio‐economic Index for Areas and the Index of Relative Socio‐economic Advantage and Disadvantage (SEIFA‐IRSAD). Lower = deciles 7–10; Higher = deciles 1–6.

^d^ BMI categories (kg/m^2^): Normal weight ≤ 24.9, Overweight = 25–29.9, Obese ≥ 30.

We found no overall significant association between T2DM and melanoma recurrence 7 years after diagnosis (adjusted hazard ratio [HR] 1.27, 95% CI 0.83–1.94, *p* = 0.27). However, BMI stratification showed that obese participants with T2DM had a significantly increased risk of recurrence within 7 years compared to their non‐diabetic counterparts (HR 1.96, 95% CI 1.02–3.74, *p* = 0.04), suggesting a potential additive effect of these comorbidities possibly due to increased or chronic inflammation (Table 3).

**TABLE 3:** Association between melanoma recurrence at 7 years post‐diagnosis and type 2 diabetes mellitus (T2DM) using a Cox proportional hazards model.

		*N* (%)	Unadjusted HR^a^ (95% CI)	*p*	Adjusted^b^ HR (95% CI)	*p*
Diabetes						
No		151 (84)	1.00 *Reference*		1.00 *Reference*	
Yes		28 (16)	1.28 (0.86–1.92)	0.23	1.27 (0.83–1.94)	0.27
Stratified Analyses						
BMI^c^						
Normal weight	No Diabetes	43 (91)			1.00 *Reference*	
	Diabetes	4 (9)			1.34 (0.44–4.09)	0.61
Overweight	No Diabetes	60 (92)			1.00 *Reference*	
	Diabetes	5 (8)			0.81 (0.31–2.10)	0.66
Obese	No Diabetes	33 (67)			1.00 *Reference*	
	Diabetes	16 (33)			1.96 (1.02–3.74)	**0.04**
Socioeconomic status^d^						
Lower	No Diabetes	74 (80)			1.00 *Reference*	
	Diabetes	18 (20)			1.52 (0.86–2.61)	0.13
Higher	No Diabetes	77 (89)			1.00 *Reference*	
	Diabetes	10 (11)			0.82 (0.40–1.69)	0.59

*Note:* Bold values indicate *p* < 0.05.

Abbreviations: BMI, body mass index; CI, confidence interval; HR, hazard ratio.

^a^ Hazard ratio for risk of recurrence within 7 years of diagnosis of the primary tumour.

^b^ Adjusted for age, sex, tumour thickness, presence of ulceration, and statin use.

^c^ BMI categories (kg/m^2^): Normal weight ≤ 24.9, Overweight = 25–29.9, Obese = ≥ 30.

^d^ Calculated using the Socio‐economic Index for Areas and the Index of Relative Socio‐economic Advantage and Disadvantage (SEIFA‐IRSAD). Lower = deciles 7–10; Higher = deciles 1–6.

Our study is limited in that T2DM diagnosis was self‐reported (confirmed using medical records). Given the heterogeneity of melanoma and T2DM, it is possible that other unexplored clinicopathological factors could have contributed to these associations.

We conclude that T2DM may not be an independent risk factor for tumour thickness or recurrence but acts in conjunction with BMI. Lower SES individuals with T2DM, compared to non‐diabetics, are more likely to have a thinner melanoma at diagnosis. Additionally, obese individuals with T2DM have a higher risk of developing a recurrence within 7 years of diagnosis compared to obese non‐diabetics.

## Ethics Statement

Reviewed and approved by the University of Queensland ID#15895. Metro South Hospital and Health Service and QIMR Berghofer Medical Research Institute ethics committees.

## Consent

The authors have nothing to report.

## Conflicts of Interest

The authors declare no conflicts of interest.

## Data Availability

The data that support the findings of this study are available from the corresponding author upon reasonable request.
